# Supplementary figures and images for: Zika virus isolation, propagation, and quantification using multiple methods
Source: PLoS One. 2021 Jul 30;16(7):e0255314. doi: 10.1371/journal.pone.0255314 (PMC8323943; doi:10.1371/journal.pone.0255314)

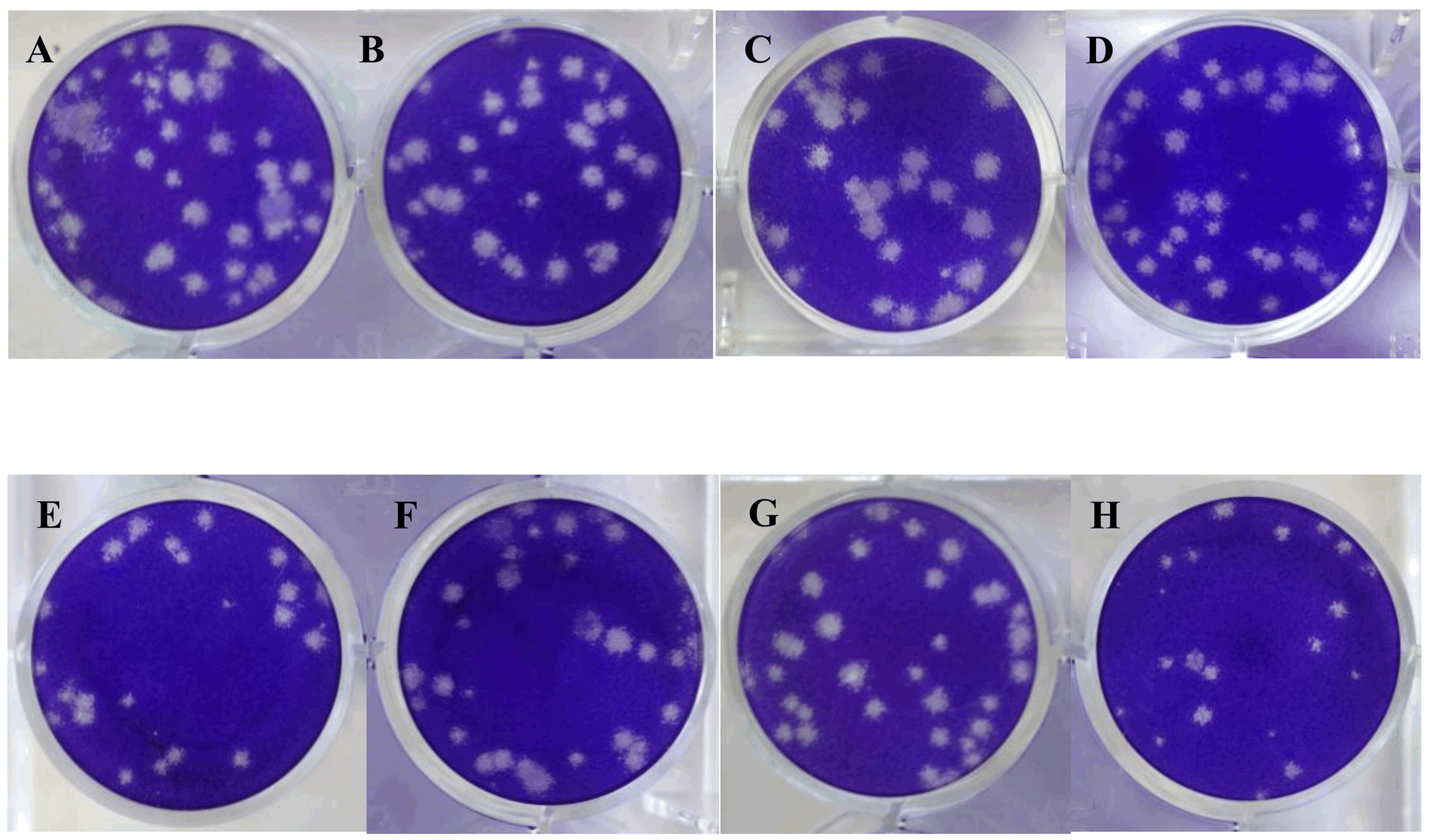

Supplement: S1 Fig — The plaque sizes of 8 ZIKV isolates; (A) MU-DMSC-2/2016, (B) MU-DMSC-3/2016, (C) MU-DMSC-4/2016, (D) MU-DMSC-5/2016, (E) MU-DMSC-2/2017, (F) MU-DMSC-3/2017, (G) MU-DMSC-4/2017 and (H) MU-DMSC-5/2017. (TIF) [file pone.0255314.s001.tif]
