# Supplementary material for: Zika virus isolation, propagation, and quantification using multiple methods
Source: PLoS One. 2021 Jul 30;16(7):e0255314. doi: 10.1371/journal.pone.0255314 (PMC8323943; doi:10.1371/journal.pone.0255314)
Supplement: Attachment — Submitted filename: Response to Reviewers_10-06-2021.docx [file pone.0255314.s002.docx]

**Response to Reviewer**

**PONE-D-21-11564**
**Article title:** Zika virus isolation, propagation, and quantification using multiple methods

Reviewers' comments:
Reviewer's Responses to Questions

**Comments to the Author**
1. Is the manuscript technically sound, and do the data support the conclusions?

The manuscript must describe a technically sound piece of scientific research with data that supports the conclusions. Experiments must have been conducted rigorously, with appropriate controls, replication, and sample sizes. The conclusions must be drawn appropriately based on the data presented.

Reviewer #1: Partly

2. Has the statistical analysis been performed appropriately and rigorously?

Reviewer #1: N/A

3. Have the authors made all data underlying the findings in their manuscript fully available?

The [PLOS Data policy](http://www.plosone.org/static/policies.action#sharing) requires authors to make all data underlying the findings described in their manuscript fully available without restriction, with rare exception (please refer to the Data Availability Statement in the manuscript PDF file). The data should be provided as part of the manuscript or its supporting information, or deposited to a public repository. For example, in addition to summary statistics, the data points behind means, medians and variance measures should be available. If there are restrictions on publicly sharing data—e.g. participant privacy or use of data from a third party—those must be specified.

Reviewer #1: Yes

4. Is the manuscript presented in an intelligible fashion and written in standard English?

PLOS ONE does not copyedit accepted manuscripts, so the language in submitted articles must be clear, correct, and unambiguous. Any typographical or grammatical errors should be corrected at revision, so please note any specific errors here.

Reviewer #1: No

**Response:** We have Dr. Arthur Brown, a native English speaker, editing our language in the revised manuscript as shown in acknowledgement.

5. Review Comments to the Author

Please use the space provided to explain your answers to the questions above. You may also include additional comments for the author, including concerns about dual publication, research ethics, or publication ethics. (Please upload your review as an attachment if it exceeds 20,000 characters)

Reviewer #1: In this manuscript “Zika virus isolation, propagation, and quantification using multiple methods” by Dangsagul et al., the authors attempted to isolate Zika virus (ZIKV) from a total of 270 specimens (92 sera, 171 urines, and 7 autopsy tissue samples) by intrathoracic inoculation of samples into Toxorhynchitis splendens mosquitoes, followed by three passages in C6/36 mosquito cells. From these 270 samples, the authors were able to isolate ZIKV from 11 samples. Isolated ZIKV were titrated by plaque (plaque forming units, PFU) and immunofluorescence (focus forming units) assays in Vero cells and viral genome copies were determined by real time RT-PCR and digital RT-PCR. The manuscripts demonstrate the feasibility of isolate ZIKV using inoculation of Toxorhynchitis splendens mosquitoes followed by passage in C6/36 mosquito cells, and that plaque and immunofluorescence assays results in similar viral titers while quantification by real-time or digital droplet RT-PCR were higher than those obtained using plaque or immunofluorescence assays.

Overall, this is a descriptive manuscript that could probably fit better in a methodology journal since provide with technical approaches for isolation and titration of ZIKV. Notably, although the authors have been able to isolate and observe differences in the plaque phenotype of the isolated ZIKV, genome sequence of the viral genomes have not been provided. The manuscript will improve if the authors provide with the sequence analysis of the different isolated ZIKV that could explain the differences in the plaque phenotype. This is particularly important because the virus with the biggest plaque phenotype (MUMT-1/2016) was isolated from a brain sample, contrary to the other ZIKV that were isolated from either urine or serum samples.
**Response:** We conducted complete genome sequencing of these 11 ZIKV isolates by the Sanger method and deposited the sequencing data in the GenBank database. The sequencing data will be released in June 2021 using the accession numbers as shown in Table 1. The information on molecular characterization of these 11 viruses together with the other ZIKV isolates in Thailand is shown in the other manuscript under reviewing process by the other publisher. In response to this important comment from the reviewer, we add more information about the genetics of these 11 ZIKV isolates in the **Materials and Methods**, and the **Discussion** of the revised manuscript as follows.

**Line Nos. 201-204:**

**Genomic characteristics of the ZIKV isolates**

Complete genome sequencing by the Sanger method showed that all 11 ZIKV isolates belonged to the Asian lineage. These sequences can be retrieved from the GenBank database using the accession numbers shown in Table 1.

**Line Nos. 276-281:**

Nevertheless, using the Sanger method for nucleotide sequencing, we did not see any double peaks in the chromatograms, a marker of a virus quasi-species. Moreover, difference in plaque sizes across the viral isolates, particularly the larger plaque size of an isolate from the brain stem, may be related to the viral virulence. We are analyzing the virulence determinants and phylogenetic relationship of our genomic sequences against the others.

There are also some minor concerns:
1) The authors could provide with plaque assays for the 11 isolated ZIKV (Table 1) rather than a selected set of 3 of them (Figure 1).

**Response:** We have included the plaque characteristics of 8 more ZIKV isolates in the S1 Fig. We have included this information in Line No. 223.

2) The authors indicate that some of the ZIKV isolates presented with viruses with different plaque sizes, suggesting the presence of mix population of viruses. However, plaque assays shown in Figure 1 suggest a similar plaque phenotype in the three isolated ZIKV MUM-1/2016 (A), MU-DMSC-1/2017 (B) and MU-DMSC-6/2016.

**Response:** The three ZIKV isolates mentioned displayed different plaque sizes, but the limitation of photograph resolution may have made the small plaque sizes not clearly visible.

We put the arrows to make the pictures clearer.

3) The authors should revise the manuscript to provide with some missing information (e.g. source of Vero cell lines) and to keep consistency with the nomenclature (e.g. ZIKV vs. Zika virus).

**Response:** The C6/36 cell line (ATCC CRL-1660) and Vero cell line (ATCC CCL-81) were used. Please see Line no.112, and 149, respectively. We replace the nomenclature of Zika virus with ZIKV.

4) Line 219 indicate Table 2 but I think the authors refer to Table 1.

**Response:** Thank you. We change “Table 2” in line 221 to “Table 1”.
