# Supplementary material for: Zika virus isolation, propagation, and quantification using multiple methods
Source: PLoS One. 2021 Jul 30;16(7):e0255314. doi: 10.1371/journal.pone.0255314 (PMC8323943; doi:10.1371/journal.pone.0255314)
Supplement: Attachment — Submitted filename: Additional Editor Comments_11-07-2021.docx [file pone.0255314.s003.docx]

**PONE-D-21-11564R1 - [EMID:d7f4c12d9bb6967e]
Article title:** Zika virus isolation, propagation, and quantification using multiple methods

**Additional Editor Comments (if provided):**

This revised version of a paper by Dangsagul and colleagues has addressed the main concerns raised by the reviewer of the original submission, with exception of the question raised by the reviewers about the plaque size phenotype.
The response provided by the authors is not satisfactory. The authors need to provide additional information about the characterization of the plaque size phenotype by confirming a stable plaque size phenotype of plaque purified viral populations exhibiting different plaque size shown in figure 1 (red and green arrows).

**Response to additional comment**

Plaque variants or the mixed plaque size phenotype are common in several virus isolates of early passages. This phenotype may be due to the difference in fitness of the individual infectious virus to grow in new hosts. Usually, the virus with a larger plaque size replicates faster and will overgrow and replace the smaller plaque size after sub-passaging. Consequently, the virus preparation will contain a homogeneous population of larger plaque size.

Virologists performed plaque purification and characterization for specific purposes, such as to study the viral virulence determinants or when the purified virus population is essential for vaccine development or molecular cloning. Plaque purification and characterization by themselves are sufficient to serve as the specific aims in an article, as exampled below.

1. Kato F, Tajima S, Nakayama E, Kawai Y, Taniguchi S, Shibasaki K, et al. Characterization of large and small-plaque variants in the Zika virus clinical isolate ZIKV/Hu/S36/Chiba/2016. Sci Rep. 2017;7(1):16160. https://doi.org/10.1038/s41598-017-16475-2.
2. Mandary MB, Masomian M, Ong SK, Poh CL. Characterization of plaque variants and the involvement of quasi-species in a population of EV-A71. Viruses. 2020;12(6):651. https://doi.org/10.3390/v12060651.
3. Jia Y, Moudy RM, Dupuis AP, Ngo KA, Maffei JG, Jerzak GV, et al. Characterization of a small plaque variant of West Nile virus isolated in New York in 2000. Virology. 2007;367(2):339-47. https://doi.org/10.1016/j.virol.2007.06.008.

Nevertheless, the objectives of our manuscript are to isolate Zika viruses from clinical specimens and determine the virus titers using multiple techniques: plaque assay, focus forming unit assay, droplet digital RT-PCR, real time RT-PCR. The plaque assay demonstrated that our Zika virus isolates were consisted of the population with mixed plaque sizes. Individual plaque, regardless of size or virulence was count as one infectious unit. Therefore, the objective of our manuscript on virus titration was fulfilled. Plaque purification and purification can take a year to finish, and the result gained does not add up much information for this manuscript. The technique should be carried out to serve a specific purpose that is more worthwhile than the virus quantification.

According to the explanation described above, we do not change the revised version of our manuscript. Please reconsider.
